# Evaluation of Male Fertility-Associated Loci in a European Population of Patients with Severe Spermatogenic Impairment

**DOI:** 10.3390/jpm11010022

**Published:** 2020-12-29

**Authors:** Miriam Cerván-Martín, Lara Bossini-Castillo, Rocío Rivera-Egea, Nicolás Garrido, Saturnino Luján, Gema Romeu, Samuel Santos-Ribeiro, IVIRMA Group, Lisbon Clinical Group, José A. Castilla, M. Carmen Gonzalvo, Ana Clavero, F. Javier Vicente, Andrea Guzmán-Jiménez, Cláudia Costa, Inés Llinares-Burguet, Chiranan Khantham, Miguel Burgos, Francisco J. Barrionuevo, Rafael Jiménez, Josvany Sánchez-Curbelo, Olga López-Rodrigo, M. Fernanda Peraza, Iris Pereira-Caetano, Patricia I. Marques, Filipa Carvalho, Alberto Barros, Lluís Bassas, Susana Seixas, João Gonçalves, Sara Larriba, Alexandra M. Lopes, Rogelio J. Palomino-Morales, F. David Carmona

**Affiliations:** 1 Departamento de Genética e Instituto de Biotecnología, Universidad de Granada, 18016 Granada, Spain; mcervan@ugr.es (M.C.-M.); lbossinicastillo@ugr.es (L.B.-C.); andreeagj@correo.ugr.es (A.G.-J.); ines.llibur@gmail.com (I.L.-B.); mburgos@ugr.es (M.B.); fjbarrio@ugr.es (F.J.B.); rjimenez@ugr.es (R.J.); 2 Instituto de Investigación Biosanitaria ibs.GRANADA, 18012 Granada, Spain; josea.castilla.sspa@juntadeandalucia.es (J.A.C.); mariac.gonzalvo.sspa@juntadeandalucia.es (M.C.G.); ana.clavero.sspa@juntadeandalucia.es (A.C.); fjvicenteprados.sspa@juntadeandalucia.es (F.J.V.); 3 Andrology Laboratory and Sperm Bank, IVIRMA Valencia, 46015 Valencia, Spain; rocio.rivera@ivirma.com; 4 IVI Foundation, Health Research Institute La Fe, 46026 Valencia, Spain; nicolas.garrido@ivirma.com; 5 Servicio de Urología, Hospital Universitari i Politecnic La Fe e Instituto de Investigación Sanitaria La Fe (IIS La Fe), 46026 Valencia, Spain; satur.lujan@ivirma.com (S.L.); gema.mag@gmail.com (G.R.); 6 IVI-RMA Lisbon, 1800-282 Lisbon, Portugal; samuel.ribeiro@ivirma.com; 7 Department of Obstetrics and Gynecology, Faculty of Medicine, University of Lisbon, 1649-028 Lisbon, Portugal; 8 Unidad de Reproducción, UGC Obstetricia y Ginecología, HU Virgen de las Nieves, 18014 Granada, Spain; 9 CEIFER Biobanco—NextClinics, 18004 Granada, Spain; 10 UGC de Urología, HU Virgen de las Nieves, 18014 Granada, Spain; 11 Instituto de Investigação e Inovação em Saúde, Universidade do Porto (I3S), 4200-135 Porto, Portugal; claudiac@ipatimup.pt (C.C.); pmarques@ipatimup.pt (P.I.M.); filipac@med.up.pt (F.C.); abarros@med.up.pt (A.B.); sseixas@ipatimup.pt (S.S.); alopes@ipatimup.pt (A.M.L.); 12 Institute of Molecular Pathology and Immunology of the University of Porto (IPATIMUP), 4200-135 Porto, Portugal; 13 Department of Pharmaceutical Sciences, Faculty of Pharmacy, Chiang Mai University, Chiang Mai 50200, Thailand; chirananK@gmail.com; 14 Laboratory of Seminology and Embryology, Andrology Service-Fundació Puigvert, 08025 Barcelona, Spain; jrsanchez@fundacio-puigvert.es (J.S.-C.); olopez@fundacio-puigvert.es (O.L.-R.); mfperaza@fundacio-puigvert.es (M.F.P.); lbassas@fundacio-puigvert.es (L.B.); 15 Departamento de Genética Humana, Instituto Nacional de Saúde Dr. Ricardo Jorge, 1649-016 Lisbon, Portugal; iris.caetano@insa.min-saude.pt (I.P.-C.); joao.goncalves@insa.min-saude.pt (J.G.); 16 Serviço de Genética, Departamento de Patologia, Faculdade de Medicina da Universidade do Porto, 4200-319 Porto, Portugal; 17 ToxOmics—Centro de Toxicogenómica e Saúde Humana, Nova Medical School, 1169-056 Lisbon, Portugal; 18 Human Molecular Genetics Group, Bellvitge Biomedical Research Institute (IDIBELL), L’Hospitalet de Llobregat, 08908 Barcelona, Spain; slarriba@idibell.cat; 19 Departamento de Bioquímica y Biología Molecular I, Universidad de Granada, 18071 Granada, Spain

**Keywords:** SNPs, genetic association analysis, impaired spermatogenesis, non-obstructive azoospermia, severe oligospermia, infertility

## Abstract

Infertility is a growing concern in developed societies. Two extreme phenotypes of male infertility are non-obstructive azoospermia (NOA) and severe oligospermia (SO), which are characterized by severe spermatogenic failure (SpF). We designed a genetic association study comprising 725 Iberian infertile men as a consequence of SpF and 1058 unaffected controls to evaluate whether five single-nucleotide polymorphisms (SNPs), previously associated with reduced fertility in Hutterites, are also involved in the genetic susceptibility to idiopathic SpF and specific clinical entities. A significant difference in the allele frequencies of *USP8*-rs7174015 was observed under the recessive model between the NOA group and both the control group (*p* = 0.0226, OR = 1.33) and the SO group (*p* = 0.0048, OR = 1.78). Other genetic associations for *EPSTI1*-rs12870438 and *PSAT1*-rs7867029 with SO and between *TUSC1*-rs10966811 and testicular sperm extraction (TESE) success in the context of NOA were observed. In silico analysis of functional annotations demonstrated cis-eQTL effects of such SNPs likely due to the modification of binding motif sites for relevant transcription factors of the spermatogenic process. The findings reported here shed light on the molecular mechanisms leading to severe phenotypes of idiopathic male infertility, and may help to better understand the contribution of the common genetic variation to the development of these conditions.

## 1. Introduction

Male infertility is considered one of the major health concerns in developed societies, affecting 10–15% of couples of childbearing age worldwide. The clinical manifestations of this condition are highly heterogeneous, as they may be influenced by physical, environmental, or genetic causes, the latter being one of the major causes [1]. Indeed, it has been reported that the two most extreme phenotypes of male infertility, i.e., severe oligospermia (SO, very low concentration of spermatozoa in semen) and non-obstructive azoospermia (NOA, complete lack of sperm in the ejaculate due to non-obstructive causes), have an important genetic component [2]. These two male infertility manifestations are characterized by severe spermatogenic impairment (SpF) and their known primary causes include different genetic alterations, such as point mutations on genes with key roles in the male gametogenesis process, Y-chromosome microdeletions, and karyotype abnormalities [3]. However, the etiology remains obscure in most SpF cases, and different pieces of evidence suggest that this idiopathic form of male infertility has a complex etiology in which common variation of the human genome, mostly single-nucleotide polymorphisms (SNPs) and copy-number variants (CNVs), may play a relevant role [1,3].

One of the most successful strategies to investigate the possible influence of common genetic variation in the development of complex traits is the genome-wide association study (GWAS) approach, in which millions of genetic polymorphisms are interrogated in a hypothesis-free fashion across the whole genome [4]. In a previous study, Kosova and colleagues [5] performed a GWAS to determine the possible causes of reduced male fertility in a study cohort composed of Hutterite men with reported fatherhood. Hutterites are a North American ethno-religious population of European descent in which contraception is proscribed, resulting in large family sizes. The authors described different genes associated with family size and several semen parameters, including *TUSC1* (MIM*610529; encoding the tumor suppressor candidate 1, which is down-regulated in non-small-cell lung cancer and small-cell lung cancer cell lines), *PSAT1* (MIM*610936; encoding a phosphoserine aminotransferase expressed in the testis), *EPSTI1* (MIM*607441; encoding the epithelial stromal interaction protein 1 highly expressed in the testis), *USP8* (MIM*603158; encoding a ubiquitin specific protein that regulates endosome morphology and it is also highly expressed in the testis), and *DPF3* (MIM*601672; encoding a transcription regulator involved in chromatin remodeling) [5].

Taking all the above into consideration, we decided to analyze for the first time whether the genetic markers of male fertility identified in the Hutterite population also conferred risk to severe spermatogenic failure (SpF), in a large cohort of Iberian men diagnosed with SO and NOA. Specific clinical entities of NOA, as well as probability of success in sperm retrieval with testicular sperm extraction (TESE) techniques, were also tested for association.

## 2. Materials and Methods

### 2.1. Study Design and Study Population

An Iberian population of 725 infertile men due to SpF (comprising 495 NOA patients and 230 SO patients) and 1058 unaffected Iberian male controls (both of European descent) were enrolled in this study. Although no principal component analyses (PCA) were performed to detect possible outliers, all participants provided a self-reported European ancestry of the Iberian Peninsula. SpF cases were recruited in different private fertility clinics as well as public centers and hospitals from Spain and Portugal. The control population included 700 population-representative healthy subjects with self-reported fatherhood as well as 358 men with normal spermatozoa number and motility, as previously described [6]. Case and control populations were matched by age, ethnicity and geographical origin (that is, all cases and controls were Iberians with European ethnicity).

Informed written consents were signed by all participants, and the procedures followed in this study were approved by the local ethical committees of every participating center, according to the tenets of the Declaration of Helsinki.

The selection criteria used to include the infertile men were based on a thorough exam of individuals showing total absence of sperm in the ejaculate (NOA) or <5 million spermatozoa/mL semen (SO) confirmed by two high-speed centrifugation processes in two different semen samples, consistent with the guidelines of the World Health Organization [7]. The medical history records were revisited to extract information related to physical examination, karyotype analysis, endocrine analysis of follicle stimulating hormone (FSH), luteinizing hormone (LH), and testosterone, as well as Y-chromosome microdeletions screening, and patients with known, genetic and non-genetic, causes of infertility were excluded from the study. In this regard, only individuals with normal karyotype, absence of Yq azoospermia factor (AZF) deletions, and a normal history of testicular development were included. In addition, those patients with a testicular biopsy performed, were classified into different subgroups according to clinical and histological data, including hypospermatogenesis (HS, extremely low numbers of mature motile sperm cells in few testicular locations), maturation arrest of germ cells (MA, >90% of maturation arrest of the germ line either at spermatogonia or at primary spermatocyte stages), and Sertoli cell-only syndrome (SCO, total absence of germ cells). Two additional subgroups were also established accordingly with the outcome in the TESE techniques (including both TESE and micro-TESE), named TESE- (including those NOA individuals in which no mature sperm cell could be retrieved from the biopsy) and TESE+ (patients with a successful sperm extraction from the biopsy). All the available information about clinical features of the patients is shown in Table S1.

### 2.2. SNP Selection and Genotyping

Three intronic variants of *USP8* (rs7174015), *DPF3* (rs10129954), and *EPSTI1* (rs12870438), as well as two intergenic variants in the regions harboring *PSAT1* (rs7867029) and *TUSC1* (rs10966811) were selected to determine their possible association with male infertility traits in our study population. The SNP selection was based on the findings by Kosova et al. [5], where they were reported to correlate with family size in a Hutterite population and with semen parameters in an independent cohort of Chicago men.

For newly recruited individuals, genomic DNA was extracted from peripheral white blood cells using the QIAamp^®^ DNA Blood Midi/Maxi (Qiagen, Hilden, Germany), Wizard^®^ Genomic DNA Purification Kit Protocol (Promega, Madison, WI, USA), or MagNA Pure LC—DNA LV Isolation kit I (Roche, Basel, Switzerland), following the procedures described by the manufacturers. The genotyping was carried out using the TaqMan^TM^ SNP genotyping technology (Applied Biosystems, Foster City, CA, USA). The real-time quantitative polymerase chain reactions (PCR) and the post-PCR allelic discriminations were performed with predesigned TaqMan^TM^ probes (assay IDs: C__26249696_10, C__31364474_20, C__32072246_20, C__30534824_10 and C___3123309_10) on a 7900HT Fast Real-Time PCR System (Applied Biosystems, Foster City, CA, USA), as described elsewhere [6].

### 2.3. Statistical Analysis

CaTS Power Calculator for Genetic Studies program [8] was used to estimate the statistical power of our study. All the statistical analyses were performed with the software Plink v1.9 [9]. Possible deviance from Hardy-Weinberg equilibrium (HWE) was evaluated in both cases and controls at the 5% significance level. To test for association between the candidate SNPs and male infertility traits, different case-control comparisons were conducted. In a first step, the whole group of SpF cases was compared against the control one. Afterwards, SpF men were divided into two different subgroups (SO and NOA) and, finally, the NOA set was further subdivided into four additional subgroups (SCO, MA, HS and TESE-). All the established case subgroups were tested against both the control group and the remaining cases not showing the specific clinical phenotype for every subgroup (in order to eliminate having NOA or SO as possible confounding variable). Allele and genotype frequencies of every tested group were compared by means of logistic regression with geographical origin (Spain or Portugal) as covariate, and assuming additive, recessive, dominant, and 2 degree of freedom (genotypic) models. *p*-values, odds ratios (ORs), and their 95% confidence intervals (CI) were then calculated, and *p*-values lower than 0.05 were considered statistically significant. Possible multiple testing effects were evaluated with the Bonferroni method.

### 2.4. In Silico Characterization of Associated Variants

Publicly available functional annotation data were explored to evaluate the possible functional implications of the observed associations using different bioinformatics tools. In a first step, we identified all the proxies (r^2^ > 0.8) of the associated lead SNPs in the overall European population (EUR) of the 1000 Genomes project phase 3 (1KGPh3) using LDLink [10]. All proxies were considered equally as candidates for prioritizing causality or hypothesizing possible underlying molecular mechanisms for the observed associations with male infertility traits. The GTEx Portal (https://www.gtexportal.org/) [11] was used to prioritize expression quantitative trait locus (eQTL) and splicing quantitative trait locus (sQTL) effects in the testis. Single-cell expression in the human testis of genes influenced by the studied SNPs was queried in the Single-Cell Expression Atlas portal (https://www.ebi.ac.uk/gxa/sc) [12]. Furthermore, we downloaded the call sets from the ENCODE portal [13] (https://www.encodeproject.org/) with the following identifiers: ENCFF323BCL, ENCFF608KRZ; ENCFF300WML, ENCFF559LDF, ENCFF644JKD, ENCFF767LMP, ENCFF788RFY, ENCFF855EVV, ENCFF286DAB, ENCFF509DBT, ENCFF316MJM, ENCFF610XSK, ENCFF819NRA, ENCFF711LHL, and ENCFF881OHS, to evaluate different regulatory chromatin marks, such as DNase-seq hypersensitivity sites, CTCF protein ChIP-seqs, H3K4me3, H3K4me1, H3K27ac, H3K9me3, and H3K27me3 histone modification ChIP-seqs. SNP-based information was also extracted from HaploReg v.4.1. [14] (https://pubs.broadinstitute.org/mammals/haploreg/haploreg.php) and SNPnexus [15] (https://www.snp-nexus.org/) to further assess the potential significance of the candidate sequence variants. These portals integrate the variant annotations from different databases, such as Ensembl, SIFT, Polyphen, CpG, Vista enhancers, miRbase, TarBase, TargetScan, miRNA Registry, snoRNA-LBME-DB, Roadmap Epigenomics, Ensembl regulatory build, RegulomeDB [16], and functional consequence predictions based on several algorithms such as: CADD, DeepSEA, EIGEN, FATHMM, fitCons, FunSeq2 GWAVA, REMM (Tables S2 and S3).

In addition, to provide an illustrative picture of the putative functional role of the tested variants, we conducted enrichment analyses of both gene ontology (GO) terms and protein-protein interactions (PPI), considering all predicted transcription factors whose binding sites (TFBS) were altered by the lead SNPs and their proxies according to position weight matrices (PWM), using the tools for that purpose of the Retrieval of Interacting Genes/Proteins (STRING) portal [17]. 

## 3. Results

This study was conducted with an appropriate overall statistical power, as shown in Table S4. No significant deviation from HWE either in cases or controls was observed (*p* < 0.05). The genotyping success rate for every analyzed SNP was over 98%, and the minor allele frequencies (MAF) of the control groups were consistent with those of both the Iberian subpopulation (IBS) and the European super population (EUR) of the 1KGPh3 [18]. All of this evidence reinforces the reliability of the generated data and the proper implementation of the methodology used.

In a first approach, we compared the allele and genotype frequencies of the five analyzed SNPs between the SpF group (which comprises all the infertile individuals of our study cohort) with those of the unaffected control population. No significant differences between them were observed under any of the tested models (Table 1).

### 3.1. Susceptibility to Non-Obstructive Azoospermia and Specific Histological Manifestations

Subsequently, we compared the NOA group and the different NOA subgroups against the unaffected control group. Significant *p*-values were observed in the analysis of the *USP8*-rs7174015 SNP frequencies of NOA cases against controls under both the additive and recessive models (P_ADD_ = 0.0402, OR = 1.18, P_REC_ = 0.0226, OR = 1.33), and a suggestive *p*-value was obtained in the genotypic model (P_GENO_ = 0.0709) (Table 1). Consistent with this, similar results were obtained when the NOA group was compared against SO samples as control group (P_ADD_ = 0.0323, OR = 1.29; P_REC_ = 0.0048, OR = 1.78; P_GENO_ = 0.0178) (Table 2). The association under the recessive model remained significant after adjustment for multiple testing (P_REC-BONF_ = 0.0242).

In addition, a trend towards association was evident for this *USP8*-rs7174015 SNP when the allele frequencies between the subgroup of NOA patients with a negative TESE outcome (TESE-) were compared against both the unaffected control group (P_ADD_ = 0.0594, OR = 1.28, P_REC_ = 0.0977, OR = 1.38) and the subgroup of NOA patients with a positive TESE outcome (TESE+, P_ADD_ = 0.0865, OR = 1.40) (Table 1 and Table 2). Finally, suggestive *p*-values were also yielded in the HS vs. no HS comparison under both the additive (P_ADD_ = 0.0727, OR = 0.64) and recessive (P_REC_ = 0.0824, OR = 0.48) models (Table 2).

The subphenotype analysis between NOA cases with and without specific histological patterns/TESE success also reached statistical significance in the analysis of the *TUSC1*-rs10966811 variant. The minor allele of such SNP showed a significant recessive risk of the HS subphenotype (P_REC_ = 0.0205, OR = 2.88). Consistent with this observation, the *TUSC1*-rs10966811 genotype frequencies were also significantly different between the NOA subgroup of patients with HS and that without this specific spermatogenic failure (P_GENO_ = 0.0295). Similarly, the comparison between TESE- vs. TESE+ NOA patients also demonstrated that this same minor allele conferred risk of an unsuccessful TESE in a recessive manner (P_REC_ = 0.0407, OR = 0.44) (Table 2).

The remaining analyzed SNPs (*DPF3*-rs10129954, *EPSTI1*-rs12870438 and *PSAT1*-rs7867029) showed no evidence of association with any of the histological patterns considered (either when the NOA subgroups were compared against the control population or in the intra-disease comparisons).

### 3.2. Susceptibility to Severe Oligospermia

A protective effect for SO predisposition was demonstrated for the minor allele of *EPSTI1*-rs12870438 in the case-control comparison under both the additive and dominant models (P_ADD_ = 0.0229, OR = 0.75, P_DOM_ = 0.0388, OR = 0.70). The genotype distribution of this SNP was considerably different (albeit not significant) between the SO group and the control one (P_GENO_ = 0.0745) (Table 1). Suggestive *p*-values were also found for *PSAT1*-rs7867029 in the SO vs. controls analysis under both the additive and dominant models (P_ADD_ = 0.0728, OR = 0.71; P_DOM_ = 0.0548, OR = 0.67) (Table 1).

On the other hand, when the SO group was compared against the NOA one (in order to detect SO-specific associations), significant differences in the allele frequencies were found for *PSAT1*-rs7867029 considering both additive and dominant effects (P_ADD_ = 0.0351, OR = 0.66; P_DOM_ = 0.0187, OR = 0.61). The genotype distributions between SO and NOA groups for this SNP also differed significantly (P_GENO_ = 0.0487) (Table 2).

No evidence of association was observed in any of the tests performed between SO versus both NOA and control groups for *DPF3*-rs10129954 or *TUSC1*-rs10966811 (Table 1 and Table 2).

### 3.3. Evaluation of Functional Annotations

We further searched for functional annotations of the 4 polymorphisms that showed significant associations with male infertility traits in this study and their proxies (r^2^ > 0.8) in the European super population (EUR) of the 1KGPh3 (Tables S5–S7). None of the lead or proxy variants were located in coding regions, CpG Islands, or miRNA target sequences according to SNPnexus [15]. Because of that, we decided to focus on other possible regulatory effects that may alter the normal gene expression levels in the testis, exploring first the transcriptome data in the GTEx project (analysis release V8) [11].

**Figure 1:**
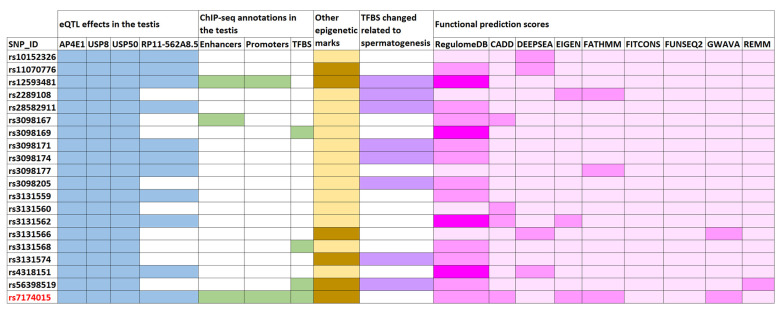
Enrichment of functional annotations of the human genome for the *USP8*-rs7174015 variant and its proxies. Overlaps are highlighted with different colors: blue for expression quantitative trait locus (eQTL) effects in the testis (affected genes are shown); green for active enhancers, active promoters, and transcription factor binding sites (TFBS) from chromatin immunoprecipitation flowing by sequencing (ChIP) experiments in the testis (using ENCODE data); orange for other epigenetic marks of the ENCODE and Roadmap Epigenomics projects (such as histone methylation and DNAase hypersensitivity); violet for TFBS modifications related to transcription factors involved in spermatogenesis based on protein weight matrix (PWM) data; and pink for functional prediction scores, in which the heatmap displays the probability of functionality for each tested variant (dark pink indicates higher probability), according to the different calculation methods described in Supplementary Tables S2 and S3.

As indicated in Figure 1, the lead SNP variant *USP8*-rs7174015 and 19 of its proxies displayed evidence of functionality in the testicular tissue as eQTL, with 11 of them affecting the expression levels of *USP8*, *USP50*, and *AP4E1*, and the remaining ones influencing also the *RP11-562A8.5* transcription levels (Figure 1). Interestingly, these four genes showed a considerable high expression in the testis according to both the Human Protein Atlas [19] (http://www.proteinatlas.org) and the GTEx database [11] (Figures S1–S4). Indeed, a testis-specific expression was evident for *USP50* and *RP11-562A8* (Figures S2 and S4). Moreover, the SNPs in this linkage disequilibrium (LD) block were also annotated as eQTLs and sQTLs in multiples tissues, including ovary (Tables S5 and S6).

**Figure 2:**
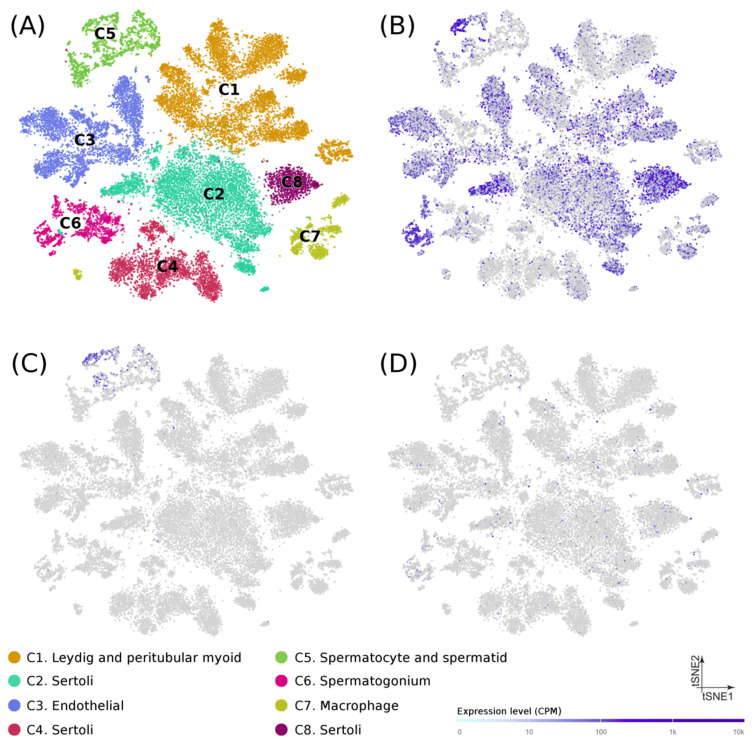
Gene expression in testicular cells from human adolescence subjects. (**A**) Dimension reduction (*t*-SNE) plots of single-cell transcriptome data in puberty human testes (*n* = 31,671) based on RNA-seq dataset from Guo et al. (Guo et al. 2020). Single cells are represented as colored dots and the different colors indicate cluster identities. Specific expression patterns of *USP8* (**B**), *UPS50* (**C**), and *AP4A1* (**D**) projected on the t-SNE plot are shown. Tonality of blue correlates with expression (with gray indicating low or no expression).

At the cellular level, recently published data from single-cell RNA-seq experiments on puberty human testes (Figure 2A) [20] showed that: (1) *USP8* was mostly expressed in spermatogonia, spermatocytes, spermatids, and Sertoli cells (Figure 2B), (2) *USP50* was detected almost exclusively in spermatocytes and spermatids (Figure 2C), and (3) *AP4E1* had a diffuse expression in multiple cell types (Figure 2D), thus suggesting a possible role of their encoded proteins in the spermatogenic process. No single-cell transcriptome data was available for *RP11-562A8*.

Moreover, six of the above mentioned linked SNPs (including *USP8*-rs7174015) overlapped with chromatin marks related to active enhancers (H3K37ac and H3K4me1), active promoters (H3K4me3), and with a TFBS of CTCF (which is involved in the conformation of the topologically associated domains) in the adult testis, according to ChIP-seq ENCODE data [13] (Figure 1 and Table S5). These variants also mapped to *loci* with several different overlapping regulatory marks in multiple tissues (including ovary) and cell lines according to Roadmap Epigenomics, ENCODE, and Ensembl Regulatory Build databases [13,21,22], thus supporting the putative regulatory relevance for this region. The output data obtained from HaploReg [14] for the *USP8*-rs7174015 LD block highlighted a large number of TFBS that were predicted to be altered by such linked SNPs based on PWM data (Table S5). We decided to prioritize them according to overlap with putative testis-specific TFBS by querying the GeneCards Suite [23] and by performing a comprehensive bibliographic search. Notably, 8 out of all the tested SNPs were predicted to change the binding motif site of transcription factors potentially involved in testicular function (Figure 1, Tables S5 and S8). For instance, rs3098174 and rs56398519 were predicted to change the TFBS of FOXJ1, a transcription factor specifically required for the formation of motile cilia and which has been reported as an important member of a pathway involved in sperm maturation in murine models [24]. Similarly, the rs3098171 SNP modified the TFBS of HSF1, a stress-inducible and DNA-binding transcription factor that plays a central role in the activation of the heat shock response (HSR), and which has been proposed essential for spermatogenesis [25]. Both rs12593481 and rs3131574 SNPs were annotated to alter the TFBS of PAX5 and NR6A1, respectively. These transcription factors have a known key role in spermatogenesis and are highly related to sperm formation and male infertility [26] (Figure 1, Tables S5 and S8). Different scores indicative of a possible functional effect of the tested variants were also calculated with tools such as RegulomeDB, CADD, deppSEA, EIGEN, FATHMM, fitCons and ReMM (Figure 1 and Table S5). Overall, both *USP8*-rs7174015 and rs12593481 showed higher scores, thus suggesting that they are the most likely causal variants of this LD block. The *USP8*-rs7174015 SNP and its proxies were also annotated as eQTLs and sQTLs in multiples tissues (Tables S5 and S6), which highlights the high relevance of this genomic region in regulatory processes. 

On the other hand, *TUSC1*-rs10966811, *EPSTI1*-rs12870438, *PSAT1*-rs7867029 and their corresponding proxies showed no significant effects on gene expression in the testis according to GTEx [11]. However, rs10812205 (a *TUSC1*-rs10966811 proxy) as well as rs58357177, rs9590722, rs9594826, and rs9594827 (all of them *EPSTI1*-rs12870438 proxies) overlapped with an open chromatin state in the testis according to ChIP-seq data from ENCODE [13], and other regulatory marks in multiple tissues. Furthermore, the SNPs rs10966813 and rs11789162 (proxies of *TUSC1*-rs10966811) were located in predicted target sequences of DMRT2 (rs10966813), DMRT7 and DMRT1 (rs11789162) according to HaploReg [14], a family of transcription factors with a key role in male sex determination and spermatogenesis [27]. The RegulomeDB score and the other functional prediction scores also suggested that the SNPs rs10812205, rs62534083, rs1535898, rs9590722, rs9594827, and rs9594829 were more likely to exert the functional effect (Table S7).

Finally, to provide a global overview of the possible pathways involved in male infertility associated with the putative causal variants, we accomplished a PPI and biological pathway enrichment analysis with 199 transcription factors that had target sequences altered by such SNPs (Tables S5 and S7). The molecular network of the selected proteins had significantly more interactions than expected (number of nodes, 98; number of edges, 459; average node degree, 9.37; clustering coefficient, 0.372; expected number of edges, 89; PPI enrichment, *p* < 1 × 10^16^, Figure S5). Regarding the functional enrichment of the network, biological processes with the highest significant *p*-values were those related to gene expression regulation processes (Table S9), consistent with the provided evidence described above. Interestingly, spermatogenesis (GO:0007283) was one of the GO terms significantly enriched in the transcription factor set (*p* = 0.0004). Indeed, some members of this biological process, such as YY1, BCL6, HOXA10, ZBTB16 (PLZF), and PAX5 (highlighted in red in Figure S5) represented relevant nodes in the PPI network.

## 4. Discussion

Idiopathic male infertility is expected to have a complex etiology likely influenced by genetic, epigenetic, and environmental factors [3]. Regarding its genetic basis, it has been estimated that the most severe expressions of this condition (NOA and SO) have a high heritability with a polygenic inheritance, in which many *loci* may exert an additive effect on the pathological phenotype [1]. In the present study, we aimed to perform the first attempt to evaluate the potential implication of five SNPs in the arising of SpF phenotype, previously associated with reduced fertility in men [5], in the largest European case-control cohort included in a genetic study to date. However, it should be noted that no PCA or determination of the human Y-chromosome haplogroup was conducted to confirm the ancestry, which represents a limitation of our study. Indeed, interactions between Y-haplogroups and autosomal variants on spermatogenic impairment, such as NOA, have been reported [28].

Our results suggest that both *EPSTI1*-rs12870438 and *PSAT1*-rs7867029 are involved in the pathological mechanisms underlying SO, whereas the intergenic SNP *USP8*-rs7174015 may contribute to the genetic susceptibility to NOA. Additionally, the minor allele of *TUSC1*-rs10966811 (A) was associated with a higher predisposition to HS-NOA subphenotype and, consequently, with a higher probability of TESE success. This observation should be highlighted, as it could help to develop reliable predictive panels for the likelihood of sperm retrieval from testicular biopsies of infertile men seeking to father a biological child, thus improving substantially the increasingly demanded counseling about the suitability of undergoing surgery in such cases [29].

Consistent with our observations, Kosova and colleagues [5] described that the risk alleles of the associated variants correlated with a decreased fertility in their study cohort. It could be speculated that the presence of such genetic variants may lead to different phenotypes related to male fertility depending on the specific genetic background of the individual, ranging from mild outcomes (such as slight reduced sperm counts or low birth rates) to more severe conditions such as SO or NOA, which supports the notion of idiopathic male infertility as a complex disease [1]. In addition, *PSAT1*-rs7867029 and *USP8*-rs7174015 were significantly associated with SO predisposition and *EPSTI1*-rs12870438 with NOA risk in a low-powered Japanese population comprising 76 NOA patients, 50 SO patients, and 791 fertile men [30]. However, the authors did not observe a correlation of such SNPs with semen parameters in an independent study cohort of Japanese males composed of 791 fertile men and 1224 young men from the general population [31]. In a subsequent study, the same group also reported significant associations of *TUSC1*-rs10966811 (associated with HS under an NOA microenvironment in our study cohort) and *DPF3*-rs10129954 (which did not yield significant *p*-values in our analyses) with SO and SpF, respectively [32]. The discrepancy of the results could be due to different genetic architectures of the regions encompassing those SNPs between Japanese and Iberian populations, or to a possible type I error affecting their results as a consequence of a reduced power (the case population included only 83 NOA patients and 62 SO patients). Indeed, for *DFP3*-rs10129954 the authors obtained significant *p*-values under opposite models (recessive and dominant) [32].

Despite the above considerations, our results clearly suggest that *TUSC1*-rs10966811 may represent a potential marker of disease outcome of NOA infertility. The *TUSC1*-rs10966811*G allele is associated with the most severe manifestation of this pathology (complete lack of sperm cells in the testis biopsy and thus TESE-), whereas the presence of the *TUSC1*-rs10966811*A allele is associated with the HS phenotype, the milder histological pattern of NOA. The functional annotations of this SNP are consistent with this idea. *TUSC1*-rs10966811 is located in a target sequence for YY1, a transcription factor that has been reported to play a major role in spermatogonial stem cell (SSC) maturation, being expressed in spermatocytes, spermatogonia, and spermatids, but not in mature spermatozoa [33,34]. The *TUSC1*-rs10966811 polymorphism represents a crucial position in the consensus sequence recognized by YY1, and the presence of the G allele correlates with a drastic decrease of the binding affinity (Table S8 and Figure S6). Other important transcription factors for the spermatogenic process have also predicted target sequences in the flanking regions of different *TUSC1*-rs10966811 proxies, such as BCL6, a repressor whose depletion causes testicular germ cell apoptosis in murine models [35]. This protein is predicted to bind the genomic region containing rs10966813, showing a lower affinity in the presence of the rs10966813*G allele, which is highly linked to *TUSC1*-rs10966811*G (the risk allele for unsuccessful TESE). In addition, DMRT proteins are a family of testis-specific transcription factors that play a pivotal role in male sex determination and differentiation by controlling testis development and male germ cell proliferation [27]. In this regard, the *TUSC1*-rs10966811 proxies rs10966813 and rs11789162 overlap with binding motifs of some members of this family, including DMRT1, DMRT2, and DMRT7. The gene encoding DMRT1 is a confirmed NOA-susceptibility *locus* [36,37,38,39], and the screening of its sequence to detect point mutations has been recently incorporated by some physicians in the routine clinical practice of idiopathic NOA to increase the diagnostic efficiency [40]. Moreover, it has been reported that open chromatin in SSCs is considerably enriched in TFBS for DMRT1 [41]. Moreover, additional transcription factors involved in spermatogenesis have also predicted binding motifs within the *TUSC1*-rs10966811 haplotype block (Table S8), suggesting that such block could have a potential interest for the development of prognostic markers of NOA.

On the other hand, our data suggest that the intergenic variant *USP8*-rs7174015*A confers risk to NOA development acting as recessive allele. This result seems consistent, as the allele frequencies were significantly different between the NOA group and both the unaffected control population and the SO group. The results of our in silico analyses were also concordant with this association. Interestingly, *USP8*-rs7174015 is annotated as an eQTL in the testis, affecting the expression of *USP8*, *USP50*, *AP4E1*, and *RP11-562A8.5*. The first of them has been reported to be highly expressed in male germ cells, in which it is involved in acrosome biogenesis [42,43]. Regarding *USP50*, *AP4E1*, and *RP11-562A8.5*, although their possible involvement in spermatogenesis has not been previously described, all three genes show a high expression in the testis [11]. Indeed, *USP50* has a testis-specific expression, mostly in spermatocytes (Figure 2). Therefore, our data suggest that *USP8*-rs7174015*A could exert its pathogenic influence in NOA predisposition by deregulating the baseline gene expression of *USP8*, *USP50*, *AP4E1*, and *RP11-562A8.5*. Such deregulation could be a consequence of an alteration of a binding protein motif by *USP8*-rs7174015*A or any of its proxies (Table S8 and Figure S7). In this context, a proxy of this SNP, rs12593481, is located within a consensus sequence for PAX5 and YY1, which are relevant transcription factors in the regulation of the spermatogenic process [33,34,44]. 

Another highly linked SNP to *USP8*-rs7174015 is rs3098171, which maps to a putative TFBS for the stress-inducible protein HSF1. The encoding gene of this transcription factor is located within the azoospermia factor b (AZFb) region of the Y-chromosome, and deletion of this region results in severe male infertility [45,46]. HSF proteins are expressed during mammalian spermatogenesis, mainly in spermatocytes and round spermatids [25]. Disruption of different HSF members, such as HSF1 and HSF2, leads to male sterility and complete lack of mature sperm in mice, as these proteins have been reported to play an essential role in the repression of sex chromatin during meiosis [47]. In this regard, the rs3098171*G risk allele, which significantly reduces the expression of the testis-specific gene *USP50*, decreases drastically the affinity of HSF1 for the TFBS in which this SNP is located. Finally, it should be also noted that, at least, 5 proxies of *USP8*-rs7174015 are annotated to map active enhancers, active promoters, and/or TFBS in the testis through ChIP-seq studies according to ENCODE [13] (Figure 1), which strongly support a putative functional implication related to their position in the genome.

In relation to the SO-associated polymorphisms *EPSTI1*-rs12870438 and *PSAT1*-rs7867029, their allele frequencies in the SO group differed from those in both the control population and the NOA group, respectively (with the two latter cohorts showing similar allele and genotype frequencies), which could be indicative of a potential implication of such SNPs in the etiology of the SpF phenotype severity. Interestingly, the rs9594826 variant, highly linked to *EPSTI1*-rs12870438, overlaps the target sequence of the transcription factor SIX5, which has been reported to decrease c-kit levels in adult mice, causing an elevated spermatogenic cell apoptosis and Leydig cell hyperproliferation [48]. In this case, a significant decrease in the binding affinity of SIX5 was also evident when the SO risk allele was present in the motif sequence (Table S8 and Figure S8).

## 5. Conclusions

In summary, we believe that our study gives an important contribution to the current knowledge about the molecular mechanisms underlying idiopathic SpF. We have evaluated the possible implication in SpF development of previously reported genetic factors associated with male fertility in a well characterized cohort of infertile men of European ancestry. Our findings may shed light on the putative role of common genetic variants in the development of specific male infertility histological patterns. Therefore, this study can contribute to a solid basis for future approaches aimed at developing more effective panels of genetic markers that could anticipate the probability of unsuccessful surgeries for retrieving viable sperm cells from the testis, which represent around half of the total surgeries currently performed in NOA patients [49]. However, additional independent and well-powered SpF cohorts may be analyzed to confirm our findings.

## Figures and Tables

**Table 1:** Analysis of the genotype and allele frequencies of the tested genetic variants comparing subgroups of clinical phenotypes of male infertility against controls.

Variant (*locus*)	1/2	Subgroup (N)	Genotype, N (%)				Additive		Recessive		Dominant		Genotypic
			1/1	1/2	2/2	MAF	*p*-Value	OR [CI 95%] *	*p*-Value	OR [CI 95%] *	*p*-Value	OR [CI 95%] *	*p*-Value
rs10129954	T/C	Controls (*n* = 1049)	220	501	328	0.4485	NA	NA	NA	NA	NA	NA	NA
(*DPF3*)		SpF (*n* = 709)	139	344	226	0.4386	0.956	1.00 [0.87–1.16]	0.700	0.95 [0.74–1.23]	0.676	1.05 [0.84–1.30]	0.782
		SO (*n* = 222)	47	96	79	0.4279	0.999	1.00 [0.80–1.25]	0.482	1.15 [0.77–1.72]	0.551	0.90 [0.64–1.27]	0.519
		NOA (*n* = 487)	92	248	147	0.4435	0.873	1.01 [0.87–1.19]	0.588	0.93 [0.70–1.22]	0.476	1.09 [0.86–1.39]	0.550
		SCO (*n* = 101)	23	51	27	0.4802	0.312	1.16 [0.87–1.55]	0.538	1.17 [0.71–1.91]	0.311	1.27 [0.80–2.01]	0.574
		MA (*n* = 51)	11	28	12	0.4902	0.265	1.26 [0.84–1.89]	0.625	1.19 [0.59–2.38]	0.207	1.54 [0.79–3.00]	0.450
		HS (*n* = 48)	7	24	17	0.3958	0.460	0.85 [0.56–1.30]	0.444	0.72 [0.32–1.65]	0.640	0.86 [0.47–1.60]	0.727
		TESE- (*n* = 140)	28	77	35	0.475	0.464	1.10 [0.86–1.40]	0.698	0.92 [0.59–1.43]	0.140	1.36 [0.90–2.03]	0.215
rs10966811	A/G	Controls (*n* = 1047)	136	520	391	0.3782	NA	NA	NA	NA	NA	NA	NA
(*TUSC1*)		SpF (*n* = 707)	97	319	291	0.3628	0.253	0.92 [0.79–1.06]	0.833	1.03 [0.77–1.39]	0.084	0.83 [0.68–1.02]	0.164
		SO (*n* = 220)	34	100	86	0.3818	0.822	0.97 [0.76–1.24]	0.538	1.16 [0.73–1.83]	0.448	0.88 [0.63–1.23]	0.502
		NOA (*n* = 487)	63	219	205	0.3542	0.191	0.90 [0.76–1.06]	0.955	0.99 [0.71–1.38]	0.078	0.82 [0.65–1.02]	0.185
		SCO (*n* = 100)	10	50	40	0.35	0.401	0.87 [0.64–1.20]	0.390	0.74 [0.38–1.47]	0.576	0.89 [0.58–1.35]	0.657
		MA (*n* = 51)	5	27	19	0.3627	0.720	0.92 [0.60–1.42]	0.491	0.72 [0.28–1.85]	0.990	1.00 [0.55–1.80]	0.773
		HS (*n* = 48)	10	17	21	0.3854	0.930	1.02 [0.66–1.58]	0.132	1.76 [0.84–3.66]	0.340	0.75 [0.41–1.36]	0.110
		TESE- (*n* = 140)	13	66	61	0.3286	0.101	0.80 [0.61–1.05]	0.220	0.69 [0.38–1.25]	0.161	0.77 [0.54–1.11]	0.262
rs12870438	A/G	Controls (*n* = 1048)	155	502	391	0.3874	NA	NA	NA	NA	NA	NA	NA
(*EPSTI1*)		SpF (*n* = 711)	101	324	286	0.3699	0.353	0.93 [0.80–1.08]	0.786	0.96 [0.72–1.28]	0.264	0.89 [0.72–1.09]	0.534
		SO (*n* = 220)	24	100	96	0.3364	**2.29 × 10^−2^**	0.75 [0.59–0.96]	0.116	0.67 [0.40–1.10]	**3.88 × 10^−2^**	0.70 [0.50–0.98]	0.074
		NOA (*n* = 491)	77	224	190	0.3849	0.924	0.99 [0.85–1.16]	0.653	1.07 [0.79–1.46]	0.641	0.95 [0.75–1.19]	0.732
		SCO (*n* = 102)	16	47	39	0.3873	0.964	0.99 [0.74–1.34]	0.831	1.06 [0.61–1.87]	0.824	0.95 [0.63–1.45]	0.932
		MA (*n* = 51)	7	23	21	0.3627	0.522	0.87 [0.57–1.33]	0.780	0.89 [0.39–2.03]	0.482	0.81 [0.45–1.45]	0.779
		HS (*n* = 48)	7	26	15	0.4167	0.615	1.12 [0.73–1.71]	0.939	0.97 [0.42–2.22]	0.441	1.28 [0.68–2.41]	0.702
		TESE- (*n* = 141)	19	64	58	0.3617	0.413	0.90 [0.69–1.16]	0.688	0.90 [0.54–1.50]	0.388	0.85 [0.60–1.22]	0.683
rs7174015	A/G	Controls (*n* = 1048)	257	541	250	0.5033	NA	NA	NA	NA	NA	NA	NA
(*USP8*)		SpF (*n* = 706)	189	351	166	0.5163	0.210	1.10 [0.95–1.27]	0.191	1.17 [0.93–1.47]	0.466	1.09 [0.86–1.39]	0.404
		SO (*n* = 221)	44	119	58	0.4683	0.380	0.90 [0.71–1.14]	0.320	0.82 [0.55–1.22]	0.662	0.92 [0.63–1.34]	0.605
		NOA (*n* = 485)	145	232	108	0.5381	**4.02 × 10^−2^**	1.18 [1.01–1.38]	**2.26 × 10^−2^**	1.33 [1.04–1.71]	0.296	1.15 [0.88–1.50]	0.071
		SCO (*n* = 102)	29	53	20	0.5441	0.213	1.21 [0.90–1.62]	0.344	1.25 [0.79–1.96]	0.282	1.32 [0.79–2.21]	0.459
		MA (*n* = 51)	16	27	8	0.5784	0.113	1.40 [0.92–2.13]	0.226	1.46 [0.79–2.71]	0.177	1.70 [0.79–3.70]	0.288
		HS (*n* = 47)	8	26	13	0.4468	0.380	0.82 [0.54–1.27]	0.320	0.67 [0.31–1.47]	0.665	0.86 [0.44–1.68]	0.606
		TESE- (*n* = 141)	44	71	26	0.5638	0.059	1.28 [0.99–1.65]	0.098	1.38 [0.94–2.03]	0.161	1.38 [0.88–2.16]	0.167
rs7867029	C/G	Controls (*n* = 1050)	15	251	784	0.1338	NA	NA	NA	NA	NA	NA	NA
(*PSAT1*)		SpF (*n* = 711)	10	155	546	0.1231	0.360	0.90 [0.73–1.12]	0.943	1.03 [0.44–2.43]	0.308	0.88 [0.70–1.12]	0.570
		SO (*n* = 221)	3	37	181	0.0973	0.073	0.71 [0.49–1.03]	0.849	0.87 [0.22–3.50]	0.055	0.67 [0.45–1.01]	0.153
		NOA (*n* = 490)	7	118	365	0.1347	0.902	0.99 [0.78–1.24]	0.967	1.02 [0.40–2.58]	0.884	0.98 [0.76–1.26]	0.987
		SCO (*n* = 103)	2	27	74	0.1505	0.542	1.14 [0.75–1.71]	0.727	1.31 [0.29–5.84]	0.569	1.14 [0.72–1.79]	0.828
		MA (*n* = 50)	1	10	39	0.12	0.673	0.87 [0.46–1.64]	0.767	1.37 [0.17–10.97]	0.586	0.83 [0.41–1.65]	0.789
		HS (*n* = 48)	1	15	32	0.1771	0.239	1.40 [0.80–2.45]	0.737	1.43 [0.18–11.52]	0.234	1.46 [0.78–2.74]	0.490
		TESE- (*n* = 141)	4	29	108	0.1312	0.910	0.98 [0.67–1.42]	0.204	2.07 [0.67–6.35]	0.620	0.90 [0.59–1.36]	0.325

* Odds ratio (OR) and 95% confidence interval (CI) for the minor allele. SpF, spermatogenic failure; NOA, non-obstructive azoospermia; SCO, Sertoli cell-only; MA, meiotic arrest; HS, hypospermatogenesis; TESE, testicular sperm extraction; SO, severe oligospermia. Significant *p*-values are highlighted in bold.

**Table 2:** Analysis of the allele and genotype frequencies of the tested genetic variants in Iberian infertile men accordingly with the presence (“with manifestation”) and the absence (“without manifestation”) of specific male infertility patterns.

Variant (*locus*)	1/2	Subgroup (N)	With Manifestation		Without Manifestation		Additive		Recessive		Dominant		Genotypic
			Genotypes (11/12/22)	MAF	Genotypes (11/12/22)	MAF	*p*-Value	OR [CI 95%] **	*p*-Value	OR [CI 95%] **	*p*-Value	OR [CI 95%] **	*p*-Value
rs10129954	T/C	SO/NOA (*n* = 222/487)	47/96/79	0.4279	92/248/147	0.4435	0.756	0.96 [0.76–1.22]	0.717	1.08 [0.71–1.63]	0.433	0.87 [0.61–1.24]	0.580
(*DPF3*)		SCO/noSCO (*n* = 101/130)	23/51/27	0.4802	25/66/39	0.4462	0.525	1.13 [0.78–1.64]	0.519	1.24 [0.65–2.34]	0.687	1.13 [0.63–2.02]	0.795
		MA/noMA (*n* = 51/180)	11/28/12	0.4902	37/89/54	0.4528	0.386	1.22 [0.78–1.91]	0.853	1.08 [0.50–2.32]	0.242	1.55 [0.74–3.24]	0.492
		HS/noHS (*n* = 48/183)	7/24/17	0.3958	41/93/49	0.4781	0.213	0.74 [0.46–1.19]	0.241	0.59 [0.24–1.43]	0.381	0.73 [0.37–1.47]	0.442
		TESE-/TESE+ (*n* = 140/92)	28/77/35	0.475	16/46/30	0.4239	0.254	1.26 [0.85–1.86]	0.622	1.19 [0.60–2.35]	0.195	1.47 [0.82–2.63]	0.429
rs10966811	A/G	SO/NOA (*n* = 220/487)	34/100/86	0.3818	63/219/205	0.3542	0.427	1.10 [0.87–1.40]	0.466	1.19 [0.74–1.92]	0.548	1.11 [0.79–1.56]	0.714
(*TUSC1*)		SCO/noSCO (*n* = 100/130)	10/50/40	0.35	15/62/53	0.3538	0.961	0.99 [0.66–1.48]	0.753	0.87 [0.37–2.04]	0.894	1.04 [0.61–1.77]	0.926
		MA/noMA (*n* = 51/179)	5/27/19	0.3627	20/85/74	0.3492	0.844	1.05 [0.65–1.70]	0.705	0.82 [0.29–2.33]	0.614	1.18 [0.62–2.26]	0.757
		HS/noHS (*n* = 48/182)	10/17/21	0.3854	15/95/72	0.3434	0.470	1.20 [0.73–1.96]	**2.05 × 10^−2^**	2.88 [1.18–7.07]	0.571	0.83 [0.43–1.59]	**2.95 × 10^−2^**
		TESE-/TESE+ (*n* = 140/92)	13/66/61	0.3286	17/37/38	0.3859	0.198	0.78 [0.53–1.14]	**4.07 × 10^−2^**	0.44 [0.20–0.97]	0.711	0.90 [0.53–1.54]	0.116
rs12870438	A/G	SO/NOA (*n* = 220/491)	24/100/96	0.3364	77/224/190	0.3849	0.126	0.83 [0.65–1.05]	0.102	0.65 [0.39–1.09]	0.321	0.84 [0.60–1.18]	0.236
(*EPSTI1*)		SCO/noSCO (*n* = 102/130)	16/47/39	0.3873	20/64/46	0.4000	0.735	0.94 [0.64–1.37]	0.917	1.04 [0.51–2.13]	0.573	0.86 [0.50–1.47]	0.814
		MA/noMA (*n* = 51/181)	7/23/21	0.3627	29/88/64	0.4033	0.519	0.86 [0.54–1.36]	0.636	0.80 [0.33–1.98]	0.567	0.83 [0.43–1.58]	0.812
		HS/noHS (*n* = 48/184)	7/26/15	0.4167	29/85/70	0.3886	0.533	1.16 [0.73–1.84]	0.774	0.88 [0.35–2.17]	0.265	1.49 [0.74–2.98]	0.419
		TESE-/TESE+ (*n* = 141/93)	19/64/58	0.3617	20/40/33	0.4301	0.169	0.77 [0.53–1.12]	0.110	0.57 [0.28–1.14]	0.436	0.81 [0.47–1.39]	0.272
rs7174015	A/G	* SO/NOA (*n* = 221/485)	44/119/58	0.4683	145/232/108	0.5381	**3.23 × 10^−2^**	0.77 [0.61–0.98]	**4.84 × 10^−3^**	0.56 [0.38–0.84]	0.519	0.88 [0.60–1.30]	**1.78 × 10^−2^**
(*USP8*)		SCO/noSCO (*n* = 102/128)	29/53/20	0.5441	33/69/26	0.5273	0.779	1.06 [0.72–1.55]	0.646	1.15 [0.64–2.06]	0.973	0.99 [0.51–1.91]	0.885
		MA/noMA (*n* = 51/179)	16/27/8	0.5784	46/95/38	0.5223	0.230	1.33 [0.84–2.11]	0.423	1.32 [0.66–2.64]	0.245	1.66 [0.71–3.90]	0.459
		HS/noHS (*n* = 47/183)	8/26/13	0.4468	54/96/33	0.5574	0.073	0.64 [0.40–1.04]	0.082	0.48 [0.21–1.10]	0.253	0.64 [0.30–1.37]	0.184
		TESE-/TESE+ (*n* = 141/91)	44/71/26	0.5638	21/46/24	0.4835	0.087	1.40 [0.95–2.04]	0.174	1.52 [0.83–2.79]	0.150	1.59 [0.85–3.00]	0.229
rs7867029	C/G	SO/NOA (*n* = 221/490)	3/37/181	0.0973	7/118/365	0.1347	**3.51 × 10^−2^**	0.66 [0.45–0.97]	0.820	1.18 [0.28–4.97]	**1.87 × 10^−2^**	0.61 [0.40–0.92]	**4.87 × 10^−2^**
(*PSAT1*)		SCO/noSCO (*n* = 103/129)	2/27/74	0.1505	2/32/95	0.1395	0.607	1.15 [0.67–1.96]	0.859	1.20 [0.16–8.71]	0.605	1.17 [0.65–2.11]	0.873
		MA/noMA (*n* = 50/182)	1/10/39	0.12	3/49/130	0.1511	0.262	0.67 [0.33–1.35]	0.784	1.38 [0.14–14.05]	0.192	0.60 [0.28–1.29]	0.366
		HS/noHS (*n* = 48/184)	1/15/32	0.1771	3/44/137	0.1359	0.486	1.25 [0.66–2.37]	0.737	1.49 [0.15–15.29]	0.505	1.27 [0.63–2.57]	0.785
		TESE-/TESE+ (*n* = 141/91)	4/29/108	0.1312	0/22/69	0.1209	0.764	1.09 [0.62–1.91]	0.999	**1.04 × 10^9^** [0.00–Inf]	0.879	0.95 [0.51–1.77]	0.864

* Odds ratios (OR) and 95% confidence intervals (CI) considering non-obstructive azoospermia (NOA) as cases and severe oligospermia (SO) as controls: additive = 1.29 (1.02–1.64), recessive = 1.78 (1.19–2.65), dominant = 1.14 (0.77–1.67). ** OR for the minor allele. MAF, minor allele frequency; SCO, Sertoli cell-only; MA, maturation arrest; HS, hypospermatogenesis; TESE, testicular sperm extraction. Significant *p*-values are highlighted in bold.

## Supplementary Materials

The following are available online at https://www.mdpi.com/2075-4426/11/1/22/s1, Table S1: Main clinical features of the 725 infertile men included in the study, Table S2: Tools for generating functional prediction scores, Table S3: RegulomeDB scoring scheme, Table S4: Estimation of the statistical power of our study for 725 patients and 1058 controls, Table S5: Overlap of the rs7174015 variant and its proxies with eQTL annotations and epigenetic marks from GTEx, ENCODE and Roadmap Epigenomic projects, Table S6: Overlap of the rs7174015 variant and its proxies with sQTL annotations from the GTEx project, Table S7: Overlap of the rs10966811, rs12870438, and rs7867029 variants and its proxies with eQTL effects and epigenetic marks from GTEx, ENCODE and Roadmap Epigenomic projects, Table S8: In silico transcription factor binding motif alterations concordantly predicted by HaploReg for SNP proxies of rs7174015, rs10966811, and rs12870438, Table S9: Enrichment of gene ontology (GO) biological processes for the set of transcription factors with changed motifs overlapping with the associated variants, Figure S1: Gene expression pattern of *USP8* in human testes, Figure S2: Gene expression pattern of *USP50* in human testes, Figure S3: Gene expression pattern of *AP4E1* in human testes, Figure S4: Gene expression pattern of *RP11-562A8.5* in human testes, Figure S5: Interaction network formed for the 199 transcription factors with predicted changed motifs by rs10966811, rs12870438, rs7174015, rs7867029 risk *loci* and their proxies, Figure S6: Sequence logos of Position Weight Matrices (PWM) for transcription factor binding sites strongly altered by *TUSC1*-rs10966811 proxies, Figure S7: Sequence logos of Position Weight Matrices (PWM) for transcription factor binding sites strongly altered by *USP8*-rs7174015 proxies, Figure S8: Sequence logos of Position Weight Matrices (PWM) for transcription factor binding sites strongly altered by *EPSTI1*-rs12870438 proxies.
