# Supplementary material for: Evaluation of Male Fertility-Associated Loci in a European Population of Patients with Severe Spermatogenic Impairment
Source: J Pers Med. 2020 Dec 29;11(1):22. doi: 10.3390/jpm11010022 (PMC7823507; doi:10.3390/jpm11010022)
Supplement: Supplementary file 1 [file jpm-11-00022-s001.zip › Hutterites_JPM_SuppFigs_R1.pdf]

Figure S1. Gene expression pattern of *USP8* in human testes. (**A**) Violin plot representation of allele-specific cis-eQTL effects on *USP8* accordingly with rs7174015 genotypes in human testicular tissue of the Genotype-Tissue Expression (GTEx, analysis release v8 and human genome build 38) database. The G and A alleles indicate the reference and alternative allele types, respectively. (**B**) RNA and protein normalized expression of *USP8* in male reproductive tissues according to the Human Protein Atlas database. (**C**) *USP8* gene expression in different human tissues of the GTEx database (analysis release v8). Testis expression is highlighted with red boxes. Teal regions indicate the density distribution of the samples and the white line in the box plots the median value of the expression.

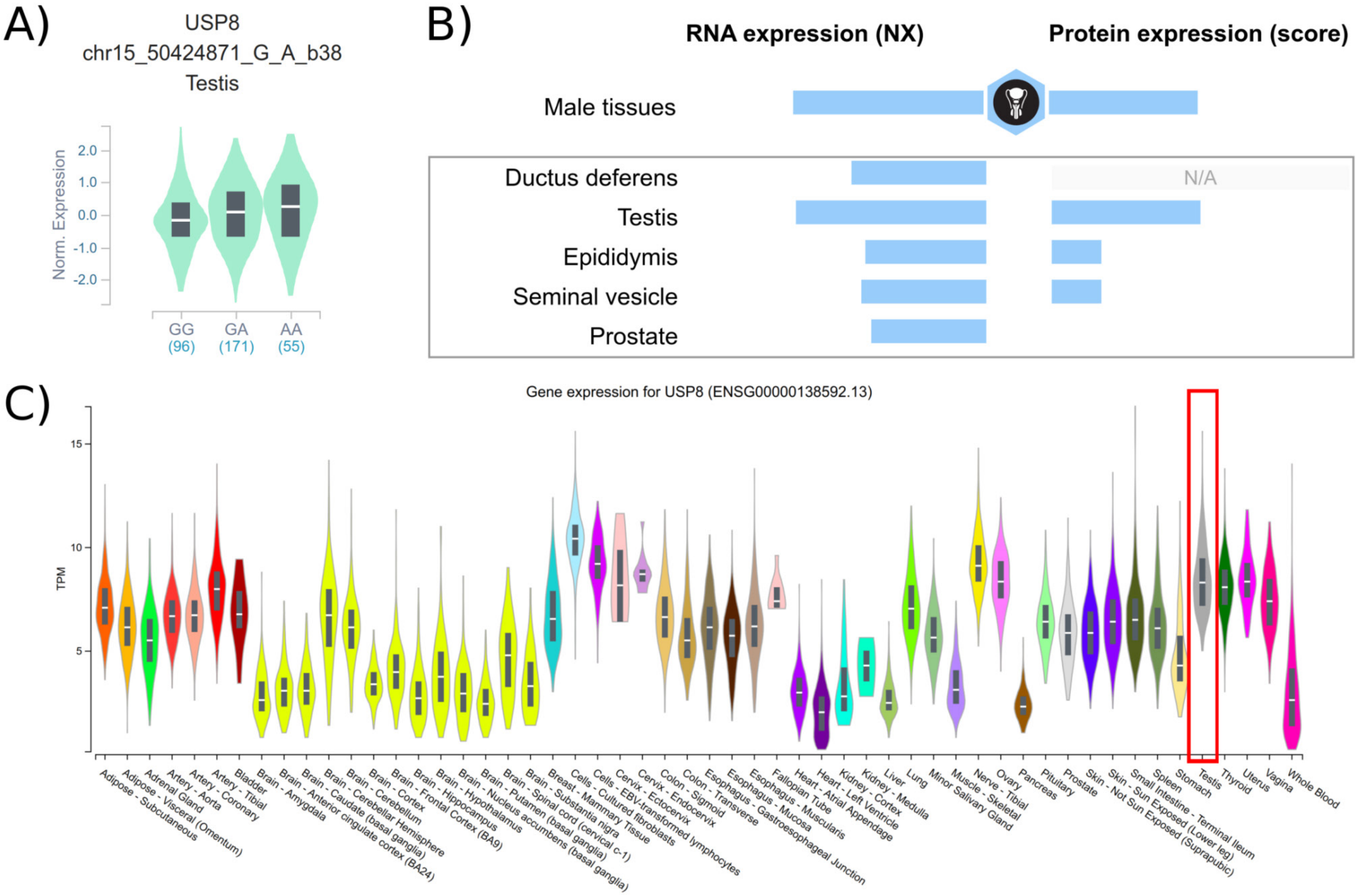

Figure S2. Gene expression pattern of *USP50* in human testes. (**A**) Violin plot representation of allele-specific cis-eQTL effects on *USP50* accordingly with rs7174015 genotypes in human testicular tissue of the Genotype-Tissue Expression (GTEx, analysis release v8 and human genome build 38) database. The G and A alleles indicate the reference and alternative allele types, respectively. (**B**) RNA normalized expression of *USP50* in male reproductive tissues according to the Human Protein Atlas database. (**C**) *USP50* gene expression in different human tissues of the GTEx database (analysis release v8). Testis expression is highlighted with red boxes. Teal regions indicate the density distribution of the samples and the white line in the box plots the median value of the expression.

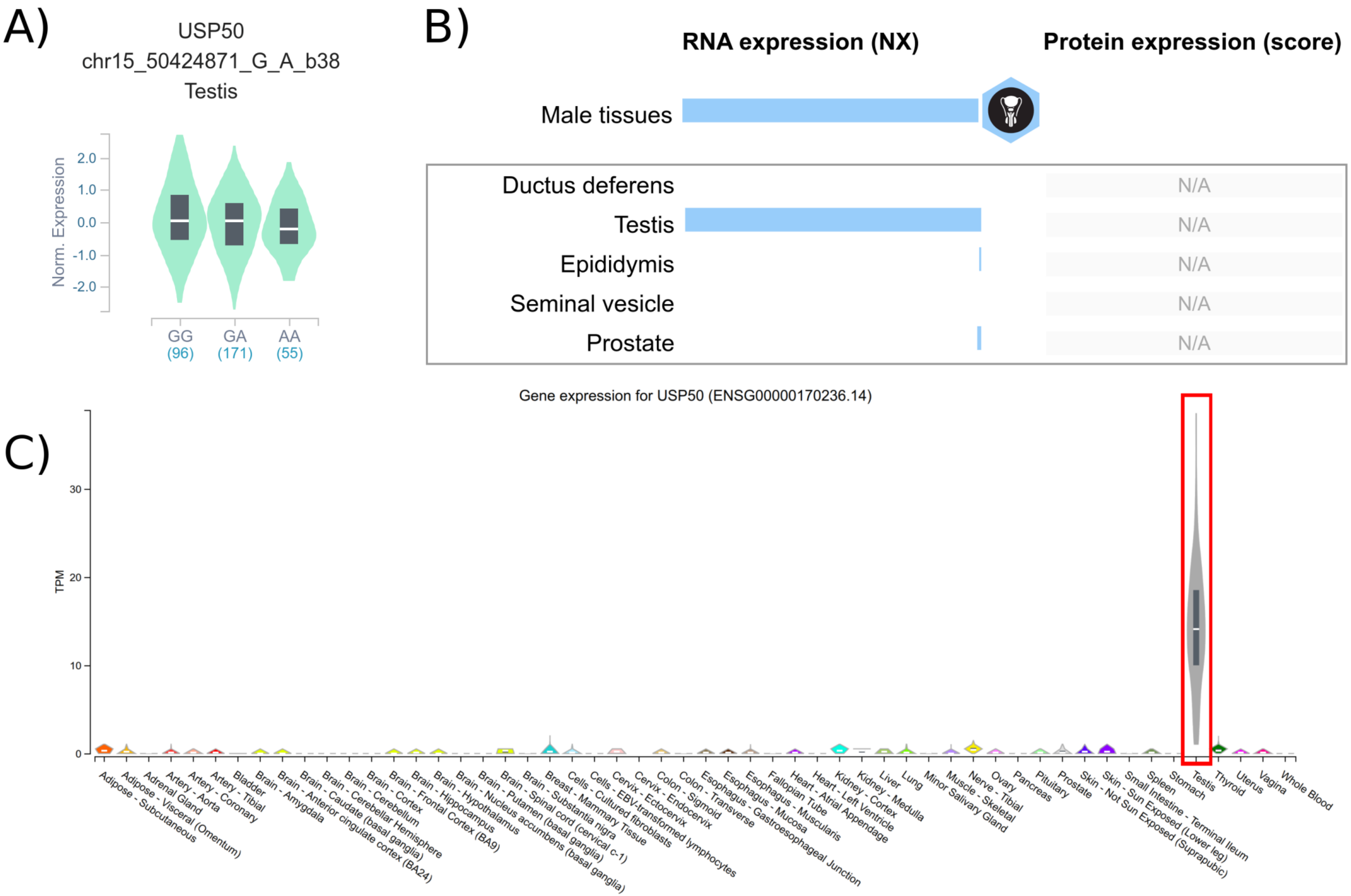

Figure S3. Gene expression pattern of *AP4E1* in human testes. (**A**) Violin plot representation of allele-specific cis-eQTL effects on *AP4E1* accordingly with rs7174015 genotypes in human testicular tissue of the Genotype-Tissue Expression (GTEx, analysis release v8 and human genome build 38) database. The G and A alleles indicate the reference and alternative allele types, respectively. (**B**) RNA and protein normalized expression of *AP4E1* in male reproductive tissues according to the Human Protein Atlas database. (**C**) *AP4E1* gene expression in different human tissues of the GTEx database (analysis release v8). Testis expression is highlighted with red boxes. Teal regions indicate the density distribution of the samples and the white line in the box plots the median value of the expression.

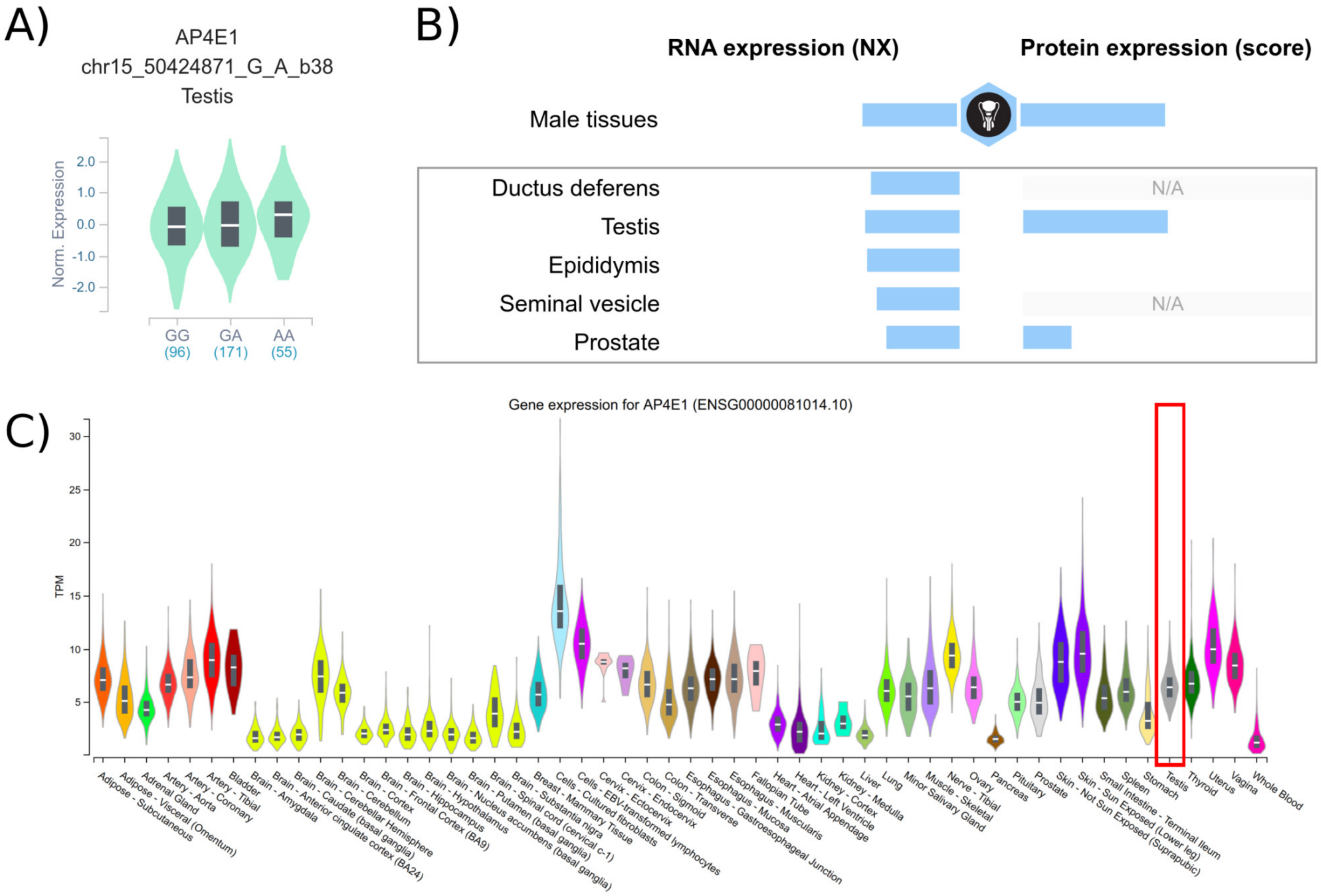

Figure S4. Gene expression pattern of *RP11-562A8.5* in human testes. (**A**) Violin plot representation of allele-specific cis-eQTL effects on *RP11-562A8.5* accordingly with rs7174015 genotypes in human testicular tissue of the Genotype-Tissue Expression (GTEx, analysis release v8 and human genome build 38) database. The G and A alleles indicate the reference and alternative allele types, respectively. (**B**) *RP11-562A8.5* gene expression in different human tissues of the GTEx database (analysis release v8). Testis expression is highlighted with red boxes. Teal regions indicate the density distribution of the samples and the white line in the box plots the median value of the expression.

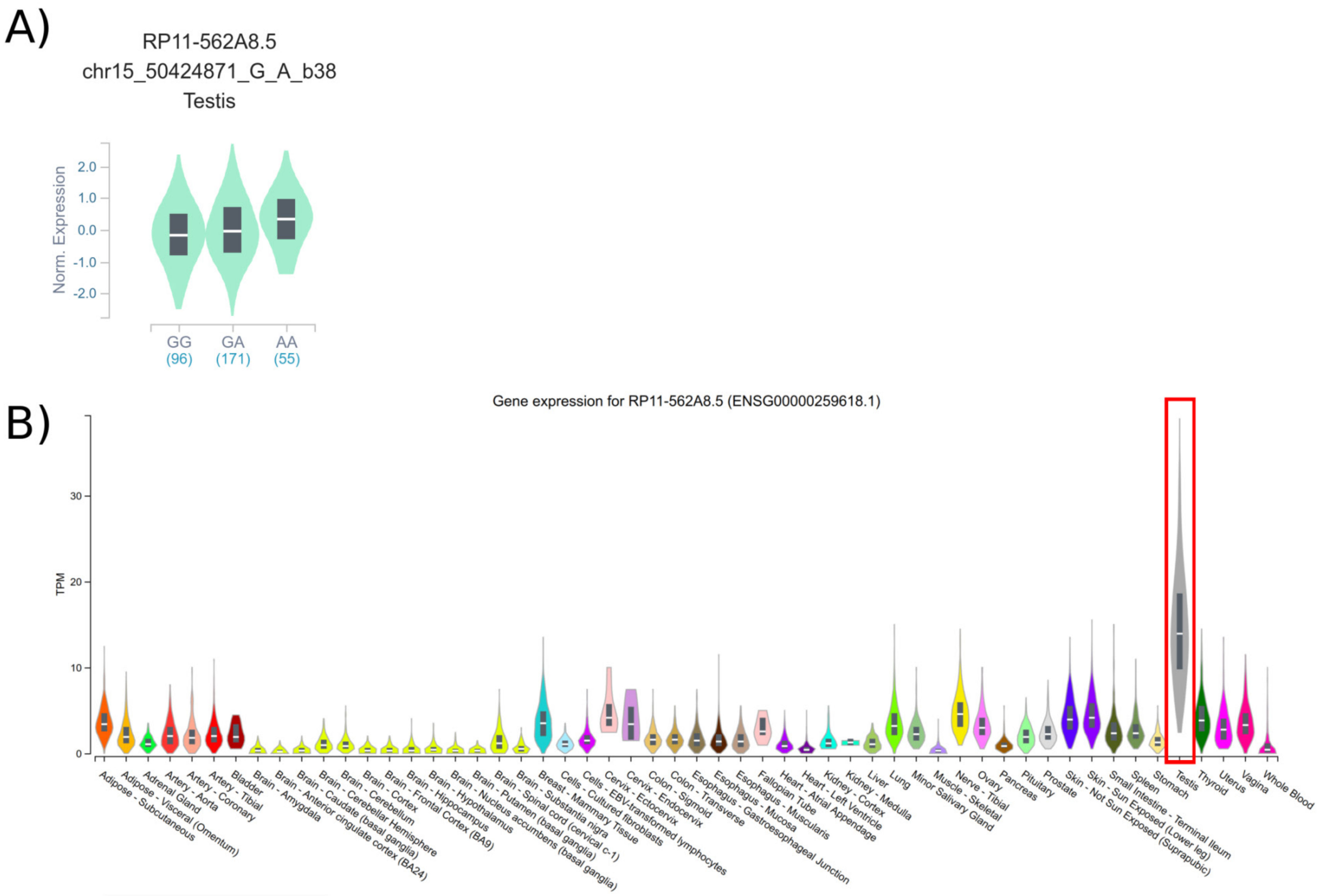

Figure S5. Interaction network formed for the 199 transcription factors with predicted changed motifs by rs10966811, rs12870438, rs7174015, rs7867029 risk *loci* and their proxies. STRING database was used to look for both direct and indirect interactions amongst selected proteins. The width of the blue lines indicates the reliability of each interaction. Transcription factors with reported specific functions in spermatogenesis are highlighted in red.

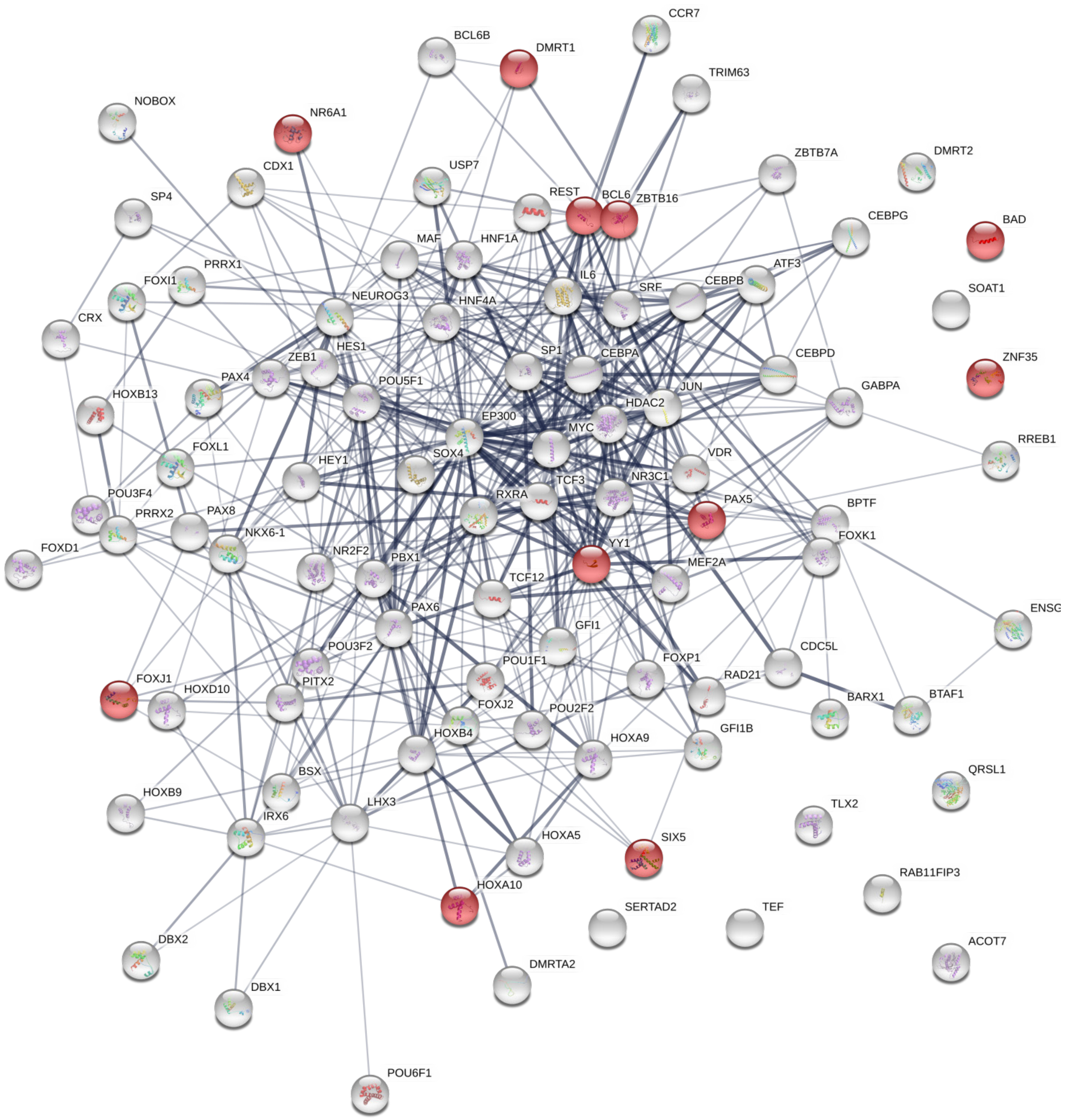

Figure S6. Sequence logos of Position Weight Matrices (PWM) for transcription factor binding sites strongly altered by *TUSC1*-rs10966811 proxies. The position of each proxy is highlighted in red for the following pairs of polymorphism/motif changed: (**A**) rs10966811/YY1_known2, (**B**) rs10966811/YY1_known6, (**C**) rs10966813/Bcl6b, (**D**) rs11789162/DMRT1.

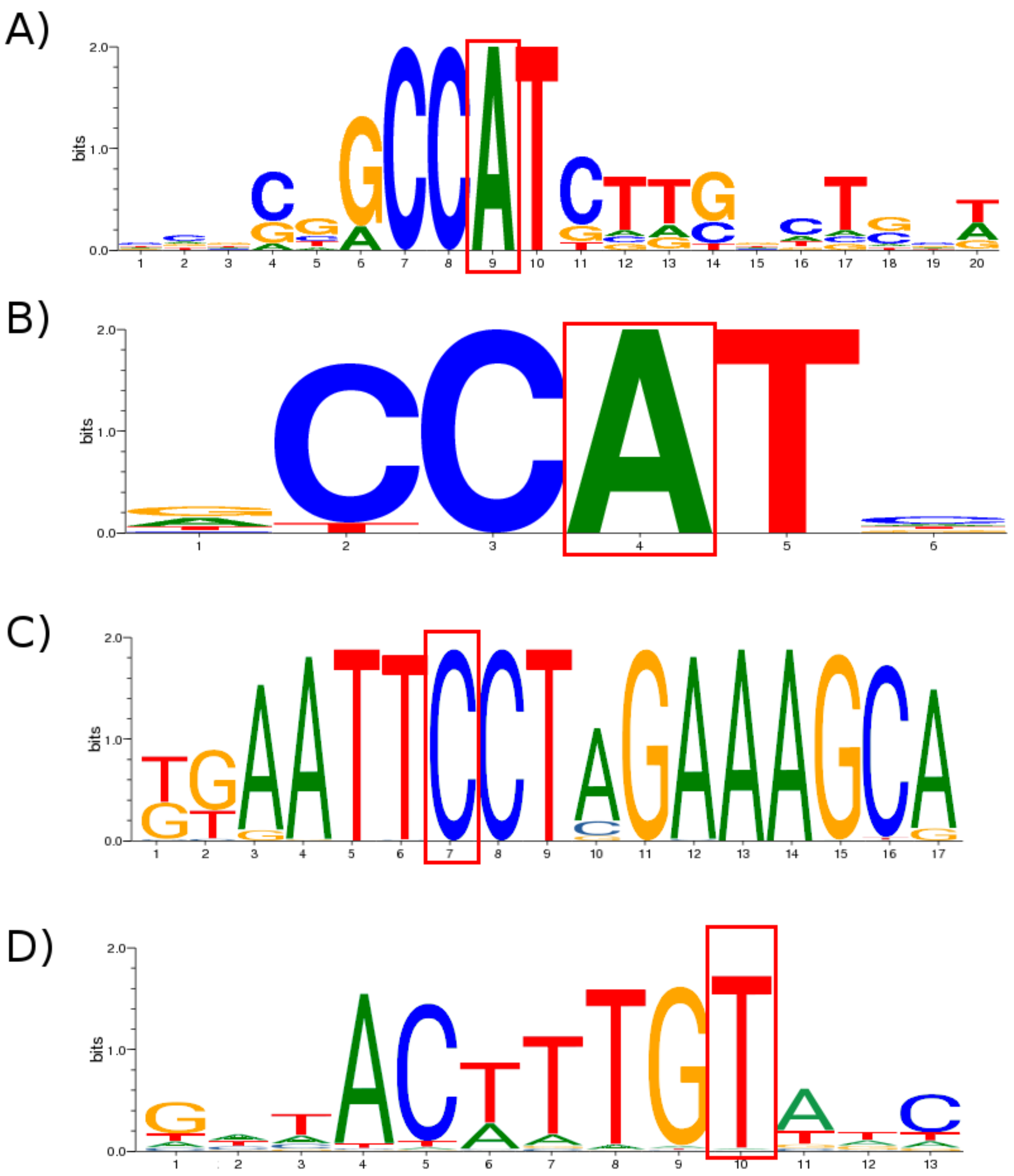

Figure S7. Sequence logos of Position Weight Matrices (PWM) for transcription factor binding sites strongly altered by *USP8*-rs7174015 proxies. The position of each proxy is highlighted in red for the following pairs of polymorphism/motif changed: (**A**) rs12593481/Pax-5_known3, (**B**) rs12593481/YY1_known4, (**C**) rs28582911/SIX5_known2, (**D**) rs3098171/Hsf_known3.

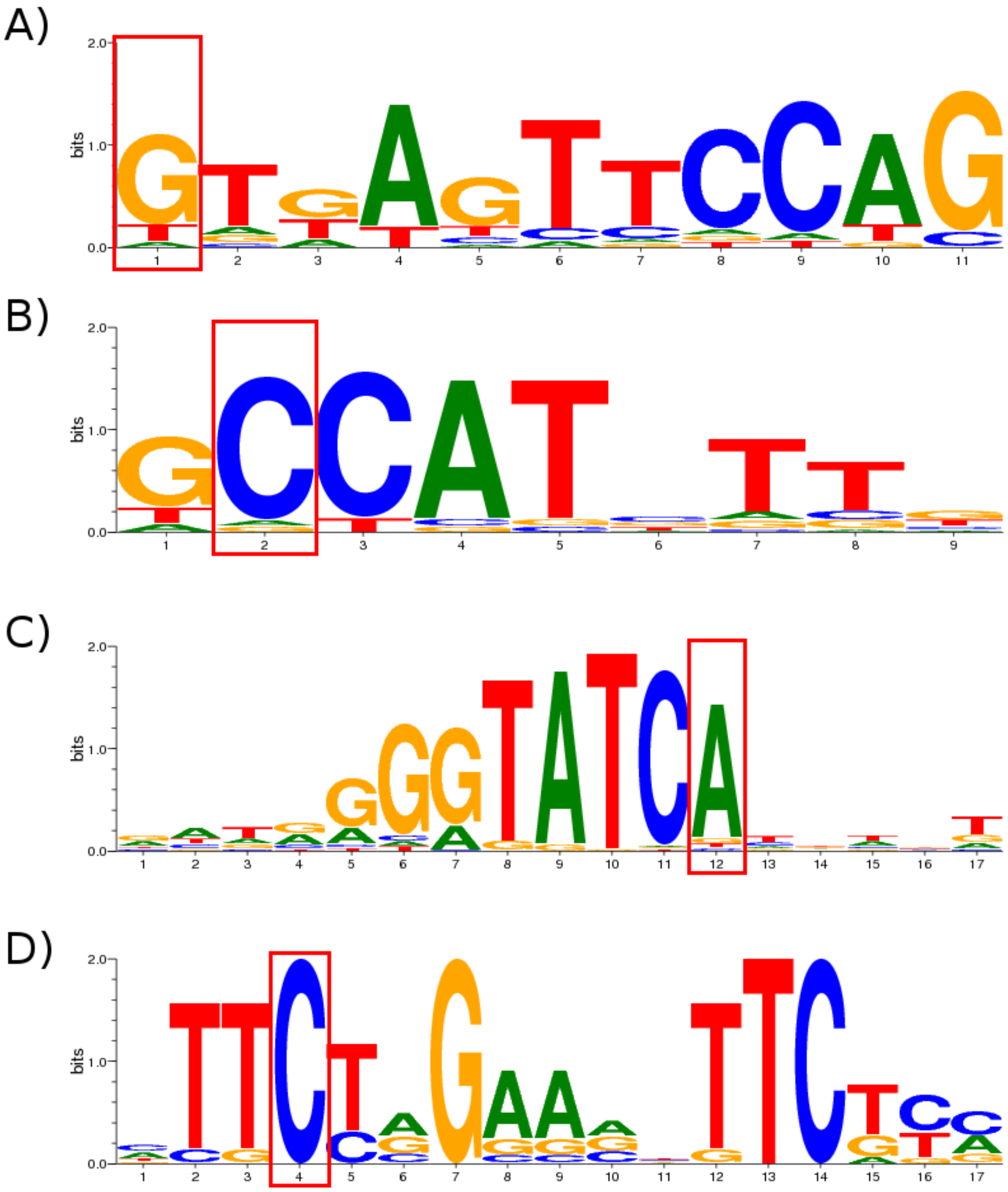

Figure S8. Sequence logos of Position Weight Matrices (PWM) for transcription factor binding sites strongly altered by *EPSTI1*-rs12870438 proxies. The position of each proxy is highlighted in red for the following pairs of polymorphism/motif changed: (**A**) rs71099806/HOXA10, (**B**) rs9594826/SIX5_known2.

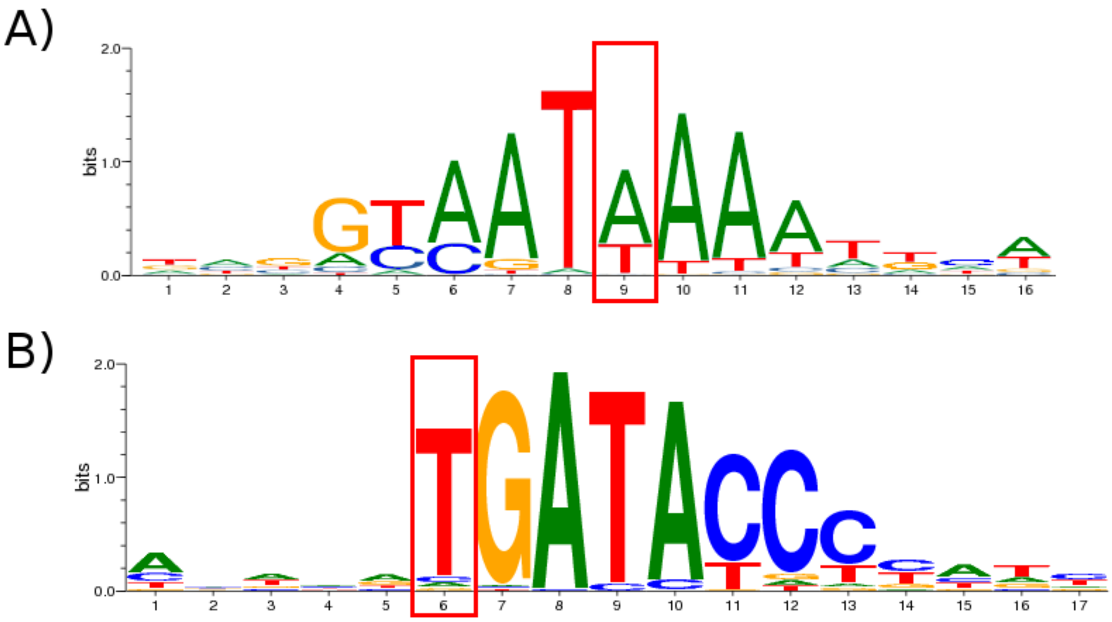

Supplementary note.

IVIRMA Group

Alberto Pacheco, IVIRMA Madrid, Spain; IVI Foundation, Health Research Institute La Fe, Valencia, Spain.

Cristina González, IVIRMA Sevilla, Spain; IVI Foundation, Health Research Institute La Fe, Valencia, Spain.

Susana Gómez, IVIRMA Lisboa, Portugal; IVI Foundation, Health Research Institute La Fe, Valencia, Spain.

David Amorós, IVIRMA Barcelona, Spain; IVI Foundation, Health Research Institute La Fe, Valencia, Spain.

Jesus Aguilar, IVIRMA Vigo, Spain; IVI Foundation, Health Research Institute La Fe, Valencia, Spain.

Fernando Quintana, IVIRMA Bilbao, Spain; IVI Foundation, Health Research Institute La Fe, Valencia, Spain.

Lisbon Clinical Group

Carlos Calhaz-Jorge, Unidade de Medicina da Reprodução, Hospital de Santa Maria, Centro Hospitalar Lisboa Norte, Lisboa, Portugal.

Ana Aguiar, Unidade de Medicina da Reprodução, Hospital de Santa Maria, Centro Hospitalar Lisboa Norte, Lisboa, Portugal.

Joaquim Nunes, Unidade de Medicina da Reprodução, Hospital de Santa Maria, Centro Hospitalar Lisboa Norte, Lisboa, Portugal.

Sandra Sousa, Unidade de Medicina da Reprodução, Hospital de Santa Maria, Centro Hospitalar Lisboa Norte, Lisboa, Portugal.

Maria Graça Pinto, Centro de Medicina Reprodutiva, Maternidade Alfredo da Costa, Centro Hospitalar Lisboa Central, Lisboa, Portugal.

Sónia Correia, Centro de Medicina Reprodutiva, Maternidade Alfredo da Costa, Centro Hospitalar Lisboa Central, Lisboa, Portugal.
